# (1*H*-Benzo­diazol-2-ylmeth­yl)di­ethyl­amine

**DOI:** 10.1107/S241431462401006X

**Published:** 2024-10-31

**Authors:** Themmila Khamrang, A. Kannan, Madhukar Hemamalini, Muhammad Nawaz Tahir, G. Jerald Maria Antony, Dhandayutham Saravanan

**Affiliations:** a Department of Chemistry Dhanamanjuri University, Manipur 795 001 India; b Department of Chemistry Anjalai Ammal Mahalingam Engineering College, Kovilvenni Tiruvarur 614 403 Tamil Nadu India; c Department of Chemistry Mother Teresa Women’s University, Kodaikanal Tamil Nadu India; d Department of Physics, University of Sargodha, Sargodha, 40100, Punjab, Pakistan; e Department of Chemistry, National College, Tiruchirappalli, Tamil Nadu, India; University of Aberdeen, United Kingdom

**Keywords:** crystal structure, benzimidazole, hydrogen bonding

## Abstract

In the crystal of the title compound, C_12_H_17_N_3_, the mol­ecules are linked by N—H⋯N hydrogen bonds, generating a *C*(4) chain extending along the *c*-axis direction. One of the ethyl groups is disordered over two sets of sites with a refined occupancy ratio of 0.582 (15):0.418 (15).

## Abstract 2

In the crystal of the title compound, the mol­ecules are linked by N—H⋯N hydrogen bonds, generating a *C*(4) chain extending along the *c*-axis direction.

## Graphical abstract

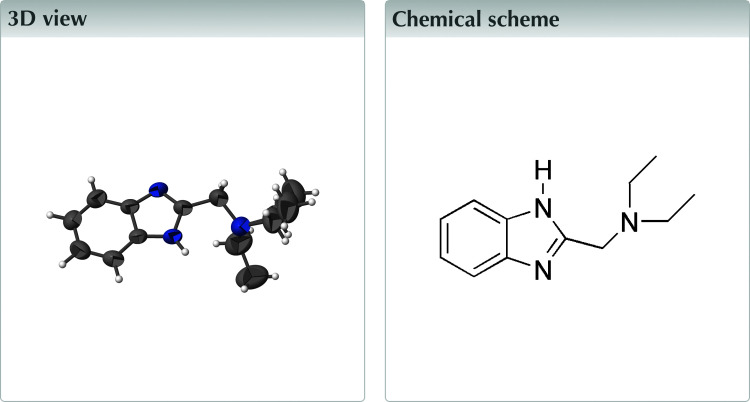

## Structure description

Benzimidazole and its derivatives show a wide range of pharmacological activities including anti­microbial, anti­fungal, anti­histaminic, anti-inflammatory, anti­viral, and anti­oxidant effects (*e.g.*, Walia *et al.*, 2011 ▸; Navarrete-Vazquez *et al.*, 2001 ▸). The present research focuses on elucidating the hydrogen-bonding patterns exhibited by the title compound, C_12_H_17_N_3_.

**Figure 1:**
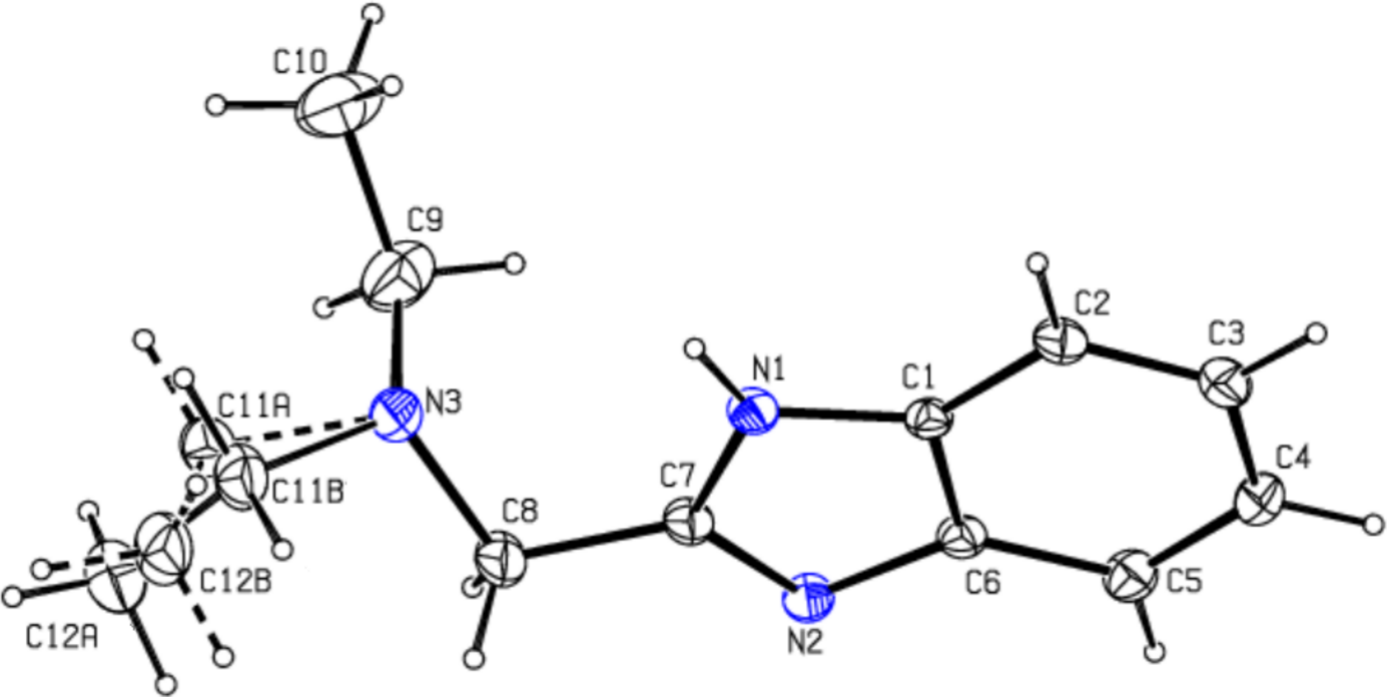
The asymmetric unit of the title compound with displacement ellipsoids drawn at the 30% probability level.

**Figure 2:**
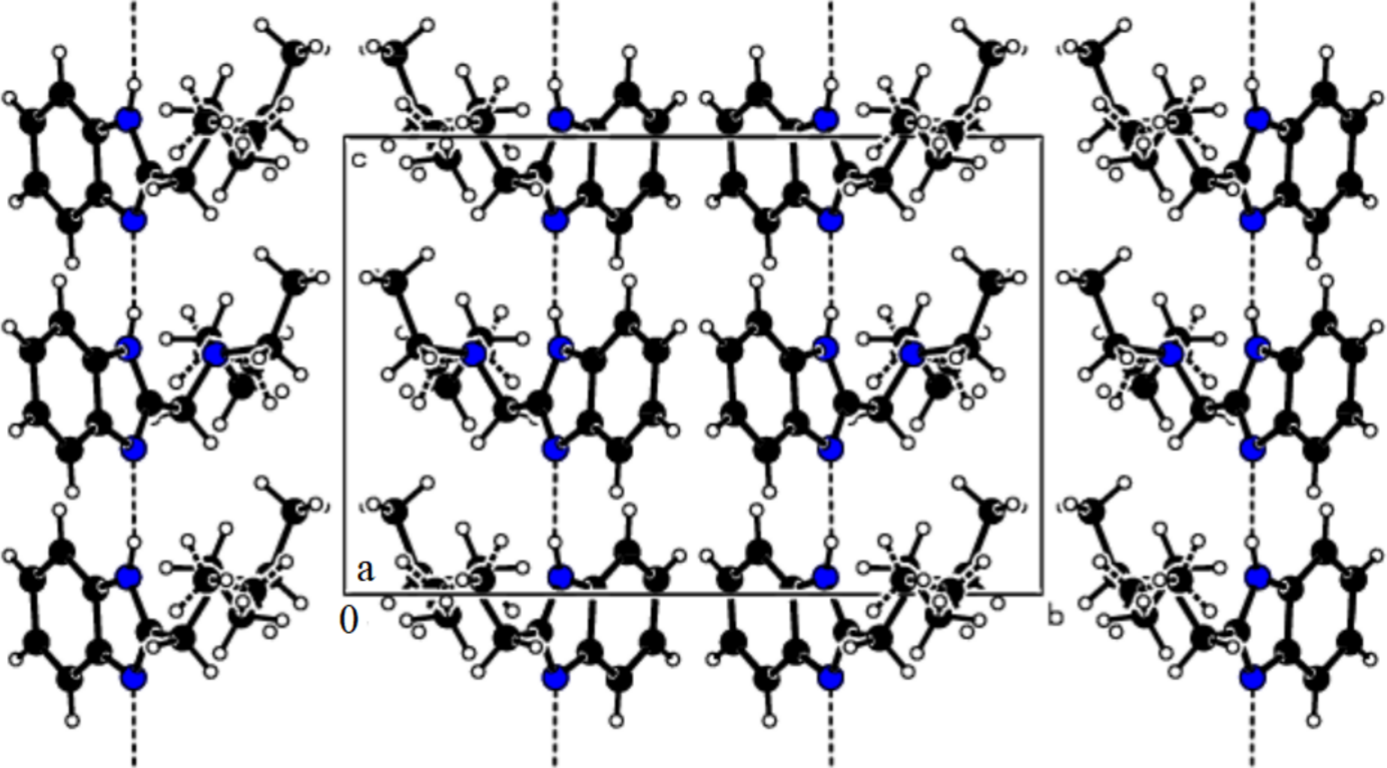
The crystal packing of the title compound.

The asymmetric unit is shown in Fig. 1 ▸. As expected, the benzimidazole (N2,N3,C6–C12) ring system is almost planar with a maximum deviation of 0.022 (8) Å for C6. The N2—C7—C8—N3 torsion angle is −155.9 (5)° and the C11/C12 ethyl group is disordered over two sets of sites with a refined occupancy ratio of 0.582 (15):0.418 (15). In the extended structure (Fig. 2 ▸), the mol­ecules are connected by N1—H1⋯N2 hydrogen bonds (Table 1 ▸) to form *C*(4) chains propagating along the *c*-axis direction.

There are thousands of benzimidazole derivatives in the Cambridge Structural Database (CSD; Version 5.43, update to November 2022; Groom *et al.*, 2016 ▸) with three examples being methyl 2-[(1*H*-benzimidazol-2-ylmeth­yl)amino]­benzoate (CSD refcode VARDEZ; Ghani *et al.*, 2011 ▸), 1-(1*H*-benzimidazol-2-yl)-*N*,*N*-bis­[(1*H*-benzimidazol-2-yl)meth­yl]methanamine methanol solvate (IHILIX; Anzaldo-Olivares *et al.*, 2020 ▸) and 1-(1-methyl-1*H*-benzimidazol-2-yl)-*N*-[(1-methyl-1*H*-benzimidazol-2-yl)meth­yl]methan­amine (TAZJIR; Gaoxiang *et al.*, 2022 ▸).

## Synthesis and crystallization

The title compound was prepared according to the literature method (Lingala *et al.*, 2011). Single crystals were obtained by slowly evaporating a di­chloro­methane solution of the title compound.

## Refinement

Crystal data, data collection and structure refinement details are summarized in Table 2 ▸.

## Supplementary Material

Crystal structure: contains datablock(s) global, I. DOI: 10.1107/S241431462401006X/hb4488sup1.cif

Structure factors: contains datablock(s) I. DOI: 10.1107/S241431462401006X/hb4488Isup2.hkl

Supporting information file. DOI: 10.1107/S241431462401006X/hb4488Isup3.cml

CCDC reference: 2376300

Additional supporting information:  crystallographic information; 3D view; checkCIF report

## Figures and Tables

**Table 1:** Hydrogen-bond geometry (Å, °)

*D*—H⋯*A*	*D*—H	H⋯*A*	*D*⋯*A*	*D*—H⋯*A*
N1—H1⋯N2^i^	0.86	2.06	2.873 (4)	157

Symmetry code: (i) 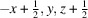.

**Table 2:** Experimental details

Crystal data	
Chemical formula	C_12_H_17_N_3_
*M*_r_	203.28
Crystal system, space group	Orthorhombic, *Pca*2_1_
Temperature (K)	293
*a*, *b*, *c* (Å)	7.9290 (7), 15.3027 (15), 10.0486 (6)
*V* (Å^3^)	1219.25 (18)
*Z*	4
Radiation type	Mo *K*α
μ (mm^−1^)	0.07
Crystal size (mm)	0.36 × 0.33 × 0.30

Data collection	
Diffractometer	Agilent Xcalibur, Atlas, Gemini
Absorption correction	Analytical (*SADABS*; Krause *et al.*, 2015 ▸)
*T*_min_, *T*_max_	0.507, 0.578
No. of measured, independent and observed [*I* > 2σ(*I*)] reflections	3304, 2135, 1138
*R*_int_	0.036
(sin θ/λ)_max_ (Å^−1^)	0.675

Refinement	
*R*[*F*^2^ > 2σ(*F*^2^)], *wR*(*F*^2^), *S*	0.066, 0.130, 1.18
No. of reflections	2135
No. of parameters	158
No. of restraints	41
H-atom treatment	H-atom parameters constrained
Δρ_max_, Δρ_min_ (e Å^−3^)	0.11, −0.10
Absolute structure	Flack *x* determined using 249 quotients [(*I*^+^)−(*I*^−^)]/[(*I*^+^)+(*I*^−^)] (Parsons *et al.*, 2013 ▸)
Absolute structure parameter	−1.1 (10)

Computer programs: *CrysAlis PRO* (Agilent, 2012 ▸), *SHELXT2014/5* (Sheldrick, 2015*a* ▸), *SHELXL2018/3* (Sheldrick, 2015*b* ▸) and *PLATON* (Spek, 2020 ▸).

## full crystallographic data

### (1H-Benzodiazol-2-ylmethyl)diethylamine Crystal data

**Table:**

C_12_H_17_N_3_	*D*_x_ = 1.107 Mg m^−^^3^
*M**_r_* = 203.28	Mo *K*α radiation, λ = 0.71073 Å
Orthorhombic, *P**c**a*2_1_	Cell parameters from 9307 reflections
*a* = 7.9290 (7) Å	θ = 3.5–26.4°
*b* = 15.3027 (15) Å	µ = 0.07 mm^−^^1^
*c* = 10.0486 (6) Å	*T* = 293 K
*V* = 1219.25 (18) Å^3^	Block, colouress
*Z* = 4	0.36 × 0.33 × 0.30 mm
*F*(000) = 440	

### (1H-Benzodiazol-2-ylmethyl)diethylamine Data collection

**Table:**

Agilent Xcalibur, Atlas, Gemini diffractometer	1138 reflections with *I* > 2σ(*I*)
Radiation source: fine-focus sealed tube	*R*_int_ = 0.036
ω scans	θ_max_ = 28.7°, θ_min_ = 3.5°
Absorption correction: analytical (SADABS; Krause *et al.*, 2015)	*h* = −10→8
*T*_min_ = 0.507, *T*_max_ = 0.578	*k* = −8→20
3304 measured reflections	*l* = −8→13
2135 independent reflections	

### (1H-Benzodiazol-2-ylmethyl)diethylamine Refinement

**Table:**

Refinement on *F*^2^	Hydrogen site location: inferred from neighbouring sites
Least-squares matrix: full	H-atom parameters constrained
*R*[*F*^2^ > 2σ(*F*^2^)] = 0.066	*w* = 1/[σ^2^(*F*_o_^2^) + (0.0388*P*)^2^] where *P* = (*F*_o_^2^ + 2*F*_c_^2^)/3
*wR*(*F*^2^) = 0.130	(Δ/σ)_max_ < 0.001
*S* = 1.18	Δρ_max_ = 0.11 e Å^−^^3^
2135 reflections	Δρ_min_ = −0.10 e Å^−^^3^
158 parameters	Absolute structure: Flack *x* determined using 249 quotients [(*I*^+^)-(*I*^-^)]/[(*I*^+^)+(*I*^-^)] (Parsons *et al.*, 2013)
41 restraints	Absolute structure parameter: −1.1 (10)
Primary atom site location: dual	

### (1H-Benzodiazol-2-ylmethyl)diethylamine Special details

**Table:**

Geometry. All esds (except the esd in the dihedral angle between two l.s. planes) are estimated using the full covariance matrix. The cell esds are taken into account individually in the estimation of esds in distances, angles and torsion angles; correlations between esds in cell parameters are only used when they are defined by crystal symmetry. An approximate (isotropic) treatment of cell esds is used for estimating esds involving l.s. planes.
Refinement. All the H atoms were positioned geometrically (C—H = 0.96–0.97 A°) and refined using a riding model with *U*_iso_(H) = 1.2 *U*_eq_(C) or 1.5 *U*_eq_(methyl C).

### (1H-Benzodiazol-2-ylmethyl)diethylamine Fractional atomic coordinates and isotropic or equivalent isotropic displacement parameters (Å^2^)

**Table:**

	*x*	*y*	*z*	*U*_iso_*/*U*_eq_	Occ. (<1)
N1	0.2389 (4)	0.3102 (2)	0.0373 (3)	0.0597 (10)	
H1	0.284732	0.301682	0.113816	0.072*	
N2	0.1987 (4)	0.3027 (3)	−0.1813 (3)	0.0659 (11)	
N3	0.5022 (5)	0.1850 (4)	0.0249 (4)	0.0951 (15)	
C1	0.0952 (5)	0.3570 (3)	0.0120 (3)	0.0515 (11)	
C2	−0.0116 (6)	0.4053 (3)	0.0930 (4)	0.0648 (13)	
H2	0.005609	0.408829	0.184357	0.078*	
C3	−0.1425 (6)	0.4474 (3)	0.0331 (5)	0.0761 (14)	
H3	−0.216070	0.480461	0.084546	0.091*	
C4	−0.1685 (6)	0.4418 (3)	−0.1041 (4)	0.0791 (15)	
H4	−0.259316	0.471219	−0.141747	0.095*	
C5	−0.0640 (6)	0.3942 (3)	−0.1845 (4)	0.0739 (14)	
H5	−0.083221	0.390253	−0.275557	0.089*	
C6	0.0716 (5)	0.3521 (3)	−0.1255 (3)	0.0570 (13)	
C7	0.2952 (5)	0.2799 (3)	−0.0819 (4)	0.0643 (12)	
C8	0.4587 (6)	0.2325 (4)	−0.0949 (4)	0.0909 (17)	
H8A	0.451582	0.191793	−0.168709	0.109*	
H8B	0.547429	0.274120	−0.114689	0.109*	
C9	0.3974 (11)	0.1078 (5)	0.0413 (6)	0.131 (2)	
H9A	0.436683	0.062760	−0.019218	0.158*	
H9B	0.282447	0.122226	0.016695	0.158*	
C10	0.3979 (10)	0.0716 (5)	0.1815 (7)	0.165 (3)	
H10A	0.386626	0.118764	0.243885	0.248*	
H10B	0.502133	0.041431	0.197381	0.248*	
H10C	0.305414	0.031777	0.192073	0.248*	
C11A	0.6923 (15)	0.1942 (10)	0.0568 (12)	0.099 (5)	0.582 (15)
H11A	0.722510	0.255582	0.058529	0.118*	0.582 (15)
H11B	0.714977	0.169826	0.144064	0.118*	0.582 (15)
C12A	0.797 (2)	0.1479 (12)	−0.0451 (15)	0.136 (6)	0.582 (15)
H12A	0.756247	0.089389	−0.056573	0.205*	0.582 (15)
H12B	0.912622	0.146040	−0.015698	0.205*	0.582 (15)
H12C	0.790966	0.178585	−0.128247	0.205*	0.582 (15)
C11B	0.6574 (15)	0.1307 (12)	0.0073 (17)	0.096 (6)	0.418 (15)
H11C	0.664688	0.106808	−0.081926	0.116*	0.418 (15)
H11D	0.662348	0.083481	0.071549	0.116*	0.418 (15)
C12B	0.793 (3)	0.1976 (13)	0.032 (3)	0.121 (8)	0.418 (15)
H12D	0.789364	0.241313	−0.036326	0.181*	0.418 (15)
H12E	0.901286	0.169560	0.031495	0.181*	0.418 (15)
H12F	0.774788	0.224638	0.117173	0.181*	0.418 (15)

### (1H-Benzodiazol-2-ylmethyl)diethylamine Atomic displacement parameters (Å^2^)

**Table:**

	*U*^11^	*U*^22^	*U*^33^	*U*^12^	*U*^13^	*U*^23^
N1	0.0612 (18)	0.085 (3)	0.0325 (16)	0.003 (2)	−0.0023 (16)	−0.0003 (19)
N2	0.074 (2)	0.093 (3)	0.0311 (17)	0.014 (2)	−0.0006 (17)	0.0026 (19)
N3	0.078 (3)	0.131 (4)	0.076 (2)	0.027 (3)	0.008 (2)	0.032 (3)
C1	0.057 (2)	0.059 (3)	0.039 (2)	−0.002 (2)	0.0025 (18)	0.004 (2)
C2	0.073 (3)	0.074 (3)	0.048 (2)	−0.001 (3)	0.010 (2)	−0.003 (2)
C3	0.075 (3)	0.078 (4)	0.075 (3)	0.010 (3)	0.021 (3)	0.003 (3)
C4	0.075 (3)	0.093 (4)	0.070 (3)	0.017 (3)	−0.003 (2)	0.020 (3)
C5	0.073 (3)	0.093 (4)	0.056 (3)	0.001 (3)	−0.008 (2)	0.008 (3)
C6	0.061 (3)	0.071 (4)	0.040 (2)	0.000 (3)	0.0020 (18)	0.004 (2)
C7	0.067 (2)	0.086 (3)	0.040 (2)	0.011 (2)	0.0071 (19)	0.001 (2)
C8	0.084 (3)	0.136 (5)	0.053 (3)	0.039 (3)	0.013 (2)	0.012 (3)
C9	0.190 (7)	0.100 (6)	0.104 (5)	0.027 (5)	0.003 (5)	0.005 (5)
C10	0.234 (9)	0.131 (6)	0.130 (6)	0.009 (6)	0.029 (6)	0.039 (5)
C11A	0.070 (8)	0.105 (12)	0.120 (10)	0.009 (10)	−0.007 (7)	0.007 (8)
C12A	0.107 (11)	0.139 (14)	0.163 (14)	0.027 (11)	0.024 (11)	0.012 (11)
C11B	0.070 (9)	0.121 (16)	0.098 (10)	0.006 (10)	0.000 (8)	0.014 (10)
C12B	0.066 (11)	0.121 (19)	0.18 (2)	−0.007 (15)	−0.020 (16)	0.022 (16)

### (1H-Benzodiazol-2-ylmethyl)diethylamine Geometric parameters (Å, º)

**Table:**

N1—C7	1.360 (5)	C8—H8A	0.9700
N1—C1	1.369 (5)	C8—H8B	0.9700
N1—H1	0.8600	C9—C10	1.513 (8)
N2—C7	1.306 (5)	C9—H9A	0.9700
N2—C6	1.379 (5)	C9—H9B	0.9700
N3—C8	1.448 (6)	C10—H10A	0.9600
N3—C9	1.453 (8)	C10—H10B	0.9600
N3—C11B	1.496 (15)	C10—H10C	0.9600
N3—C11A	1.547 (13)	C11A—C12A	1.498 (10)
C1—C2	1.388 (5)	C11A—H11A	0.9700
C1—C6	1.396 (4)	C11A—H11B	0.9700
C2—C3	1.362 (6)	C12A—H12A	0.9600
C2—H2	0.9300	C12A—H12B	0.9600
C3—C4	1.396 (6)	C12A—H12C	0.9600
C3—H3	0.9300	C11B—C12B	1.505 (11)
C4—C5	1.367 (6)	C11B—H11C	0.9700
C4—H4	0.9300	C11B—H11D	0.9700
C5—C6	1.387 (5)	C12B—H12D	0.9600
C5—H5	0.9300	C12B—H12E	0.9600
C7—C8	1.491 (6)	C12B—H12F	0.9600
			
C7—N1—C1	106.7 (3)	N3—C9—C10	113.6 (6)
C7—N1—H1	126.7	N3—C9—H9A	108.8
C1—N1—H1	126.7	C10—C9—H9A	108.8
C7—N2—C6	105.3 (3)	N3—C9—H9B	108.8
C8—N3—C9	111.5 (5)	C10—C9—H9B	108.8
C8—N3—C11B	112.1 (7)	H9A—C9—H9B	107.7
C9—N3—C11B	91.9 (7)	C9—C10—H10A	109.5
C8—N3—C11A	111.0 (6)	C9—C10—H10B	109.5
C9—N3—C11A	127.4 (7)	H10A—C10—H10B	109.5
N1—C1—C2	132.6 (4)	C9—C10—H10C	109.5
N1—C1—C6	105.6 (3)	H10A—C10—H10C	109.5
C2—C1—C6	121.8 (4)	H10B—C10—H10C	109.5
C3—C2—C1	117.2 (4)	C12A—C11A—N3	111.0 (13)
C3—C2—H2	121.4	C12A—C11A—H11A	109.4
C1—C2—H2	121.4	N3—C11A—H11A	109.4
C2—C3—C4	121.3 (4)	C12A—C11A—H11B	109.4
C2—C3—H3	119.3	N3—C11A—H11B	109.4
C4—C3—H3	119.3	H11A—C11A—H11B	108.0
C5—C4—C3	121.8 (5)	C11A—C12A—H12A	109.5
C5—C4—H4	119.1	C11A—C12A—H12B	109.5
C3—C4—H4	119.1	H12A—C12A—H12B	109.5
C4—C5—C6	117.7 (4)	C11A—C12A—H12C	109.5
C4—C5—H5	121.1	H12A—C12A—H12C	109.5
C6—C5—H5	121.1	H12B—C12A—H12C	109.5
N2—C6—C5	130.4 (3)	N3—C11B—C12B	101.0 (16)
N2—C6—C1	109.5 (4)	N3—C11B—H11C	111.6
C5—C6—C1	120.2 (4)	C12B—C11B—H11C	111.6
N2—C7—N1	113.0 (3)	N3—C11B—H11D	111.6
N2—C7—C8	124.9 (3)	C12B—C11B—H11D	111.6
N1—C7—C8	121.9 (4)	H11C—C11B—H11D	109.4
N3—C8—C7	112.2 (3)	C11B—C12B—H12D	109.5
N3—C8—H8A	109.2	C11B—C12B—H12E	109.5
C7—C8—H8A	109.2	H12D—C12B—H12E	109.5
N3—C8—H8B	109.2	C11B—C12B—H12F	109.5
C7—C8—H8B	109.2	H12D—C12B—H12F	109.5
H8A—C8—H8B	107.9	H12E—C12B—H12F	109.5
			
C7—N1—C1—C2	−176.9 (4)	C6—N2—C7—C8	−174.1 (5)
C7—N1—C1—C6	0.1 (5)	C1—N1—C7—N2	−0.6 (5)
N1—C1—C2—C3	177.3 (4)	C1—N1—C7—C8	174.5 (4)
C6—C1—C2—C3	0.7 (6)	C9—N3—C8—C7	73.2 (6)
C1—C2—C3—C4	0.2 (7)	C11B—N3—C8—C7	174.5 (8)
C2—C3—C4—C5	−0.1 (8)	C11A—N3—C8—C7	−138.7 (6)
C3—C4—C5—C6	−0.9 (7)	N2—C7—C8—N3	−155.9 (5)
C7—N2—C6—C5	178.8 (4)	N1—C7—C8—N3	29.6 (7)
C7—N2—C6—C1	−0.7 (5)	C8—N3—C9—C10	−162.1 (5)
C4—C5—C6—N2	−177.7 (5)	C11B—N3—C9—C10	83.2 (9)
C4—C5—C6—C1	1.8 (7)	C11A—N3—C9—C10	56.2 (10)
N1—C1—C6—N2	0.4 (5)	C8—N3—C11A—C12A	−68.0 (13)
C2—C1—C6—N2	177.8 (4)	C9—N3—C11A—C12A	73.8 (14)
N1—C1—C6—C5	−179.2 (4)	C8—N3—C11B—C12B	83.4 (15)
C2—C1—C6—C5	−1.8 (7)	C9—N3—C11B—C12B	−162.5 (14)
C6—N2—C7—N1	0.8 (5)		

### (1H-Benzodiazol-2-ylmethyl)diethylamine Hydrogen-bond geometry (Å, º)

**Table:**

*D*—H···*A*	*D*—H	H···*A*	*D*···*A*	*D*—H···*A*
N1—H1···N2^i^	0.86	2.06	2.873 (4)	157

Symmetry code: (i) −*x*+1/2, *y*, *z*+1/2.
